# Resonant Enhancement of Photolysis through Plasmon-Vibrational Coupling

**DOI:** 10.1021/jacs.5c20841

**Published:** 2026-01-22

**Authors:** Woo Je Chang, Logan J. Carr, Sebastian Montillo Vega, Priyansh Vora, Pengfei Huo, Delia J. Milliron

**Affiliations:** † McKetta Department of Chemical Engineering, 12330 University of Texas at Austin, Austin, Texas 78712, United States; ‡ Department of Chemistry, 12330 University of Texas at Austin, Austin, Texas 78712, United States; § Department of Chemistry, 6927 University of Rochester, Rochester, New York 14627, United States; ∥ Department of Chemical Engineering, 1259 University of Michigan, Ann Arbor, Michigan 48109, United States

## Abstract

The coupling of photonic modes and molecular vibrations is an emerging method for modifying the chemical reactivity of target molecules. However, since the photonic mode typically arises from a Fabry–Pérot cavity, it presents challenges for the systematic investigation of chemical reaction rates that require external stimuli due to the inherent closed nature of the cavity. Here, we use infrared-plasmonic colloidal nanocrystals (NCs) as building blocks in monolayer assemblies, which act as metasurfaces. We harness their strong local electric fields for coupling with molecular vibrations, allowing for control over the molecular reactivity through plasmon-vibrational coupling. After the assembly of the metasurface, we attached azidobenzoic acid to tin-doped indium oxide NCs, enabling the study of UV-driven photolysis. The photolysis rate varies when changing the frequency detuning between the azide asymmetric stretch and the collective plasmon resonance of the NC monolayer, which is controlled by the Sn doping concentration. We observe the greatest rate enhancement when the detuning approaches zero. We developed a theoretical model showing that the rate enhancement arises from vibrational excitation in the excited state of the molecules, assisted by the plasmon resonance of the NC assembly. We anticipate that our platform can serve as a scalable metasurface for controlling diverse chemical reactions and transport phenomena.

## Graphical abstract

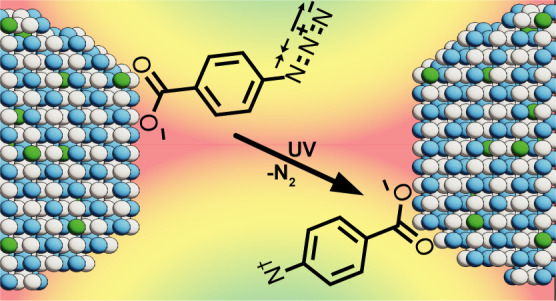

## 1 Introduction

Coupling molecular vibrations to optical modes offers a new pathway to modulate chemical reactivity without altering the reactant molecular structure itself.^1–5^ This process is mediated by the interactions between the strong electric field of the optical mode and the vibrational modes of molecules, where the coupling can influence the reaction rate and has been hypothesized to modify the reaction pathway along the potential energy surface in some cases.^6–8^ These phenomena are typically studied in optical cavities, such as Fabry–Pérot cavities, where two metallic mirrors confine resonant photonic modes that interact with vibrational modes of molecules in the cavity and modify their chemical reactivity.^9–11^ However, the inherently enclosed geometry of these cavities limits precise control and straightforward characterization of the chemical processes taking place inside.^12,13^ Despite notable recent theoretical advances,^14–20^ a broadly accepted mechanistic understanding of these vibrational strong coupling (VSC) induced effects remains elusive.^21,22^

Plasmonic metasurfaces, periodic structures of metallic nanoarrays, possess open architectures that can address the above challenges due to their “open” environment by enabling the confined electric field to directly interact with molecules or materials not necessarily enclosed. These metasurfaces convert photons into strong plasmon resonances, and the resulting intense near-field enhancement has been used to modify the electronic transitions of molecules adjacent to the metallic nanostructures and to alter the molecules’ photochemistry.^23,24^ More recently, plasmonic metasurfaces have been employed to induce vibrational coupling with molecules.^25^ This approach has been adopted to modulate the kinetics of a solvolysis reaction and the onset temperature for a dehydration reaction involving molecules engaged in vibrational interactions.^5,26^ While metasurfaces address the accessibility limitations of cavity-based designs, they are typically fabricated using electron-beam lithography, which is not easily scalable due to the high cost and complexity of the required instrumentation. In addition, tuning of the metasurface resonant modes requires changing the geometry of the patterned metallic nanostructures, which complicates some aspects of the mechanistic analysis.

To create more readily tunable and scalable metasurfaces, colloidal nanocrystals (NCs) can be used as “meta-atoms” to fabricate metasurfaces by chemical synthesis and self-assembly.^27–33^ These NCs offer strong electric field enhancement that supports effective excitonic^34^ or vibrational coupling^35^ and thus enables metasurface functionality. Colloidal NC metasurfaces are typically made from metallic NCs, and molecules located between the NCs may experience vibrational coupling.^35^ Theoretical studies have predicted that molecules confined between NCs and metal surfaces can experience significantly higher electric field densities than those in Fabry-Pérot cavities.^36^ This enhanced near-field supports the potential for NC metasurfaces to control chemical reactivity through vibrational coupling through mechanisms distinct from those envisioned for Fabry–Pérot cavities.^37^ However, the conventional metallic nature of these NCs leads to strong light–matter interactions in both the UV–visible and mid-infrared (IR) ranges, constraining the types of reactions that can be studied. As a result, photolysis and other light-driven processes in the presence of vibrational coupling remain underexplored.

Assembled plasmonic metal oxide NCs offer a promising alternative for designing colloidal NC-based metasurfaces capable of vibrational coupling owing to their IR-tunable plasmonic response. Unlike conventional metals, these wide bandgap metal oxides are transparent in the UV–visible range,^38^ leaving these wavelengths available for optical characterization or light-driven chemical reactions. Meanwhile, the localized surface plasmon resonance (LSPR) of metal oxide NCs is variable across the IR based on the dopant concentration, which provides tunability useful for probing coupling trends and mechanisms. These NCs are synthesized via bottom-up chemical methods, with size control and variable dopant concentrations readily introduced during synthesis.^39^ When assembled into a monolayer, the individual LSPR modes couple to form a collective plasmon resonance (CPR) as a metasurface, which generates strong electric field confinement within the gaps between the NCs.^27,28^ Due to the intense electric field concentration in the gaps, molecules in these regions can couple to the CPR, resulting in an increased vibrational intensity.^40–43^ This phenomenon, known as surface-enhanced infrared absorption (SEIRA), can be optimized by adjusting both the doping concentration and NC size to strengthen the coupling.^41^ We hypothesized that this coupling between the CPR mode and the vibrational modes of molecules embedded in the metasurface could also modulate the chemical reactivity.

Here, we show that molecules adsorbed on the surface of tin-doped indium oxide (ITO) NC monolayers undergo resonantly enhanced UV-driven photolysis through coupling between their molecular vibrations and the CPR of the NC monolayers. By tuning the NC size and Sn doping concentration, we established both on- and off-resonance conditions for CPR–vibration interactions and observed the highest photodegradation rate under the on-resonance condition. We further demonstrate that this coupling arises from a high-loss cavity (the CPR) and near-field coupling, differing from reaction rate modulations observed under strong coupling conditions in conventional Fabry–Pérot microcavities. Our results are consistent with the theory of resonant vibrational excitation, in which coupling to the CPR promotes the excitation of the vibrational degrees of freedom, thereby accelerating the reaction without modifying the potential energy surface. These findings demonstrate that even in the absence of vibrational strong coupling or optical excitation of plasmon modes, NC-based metasurfaces can serve as effective resonant promoters of bond cleavage under photoexcitation, thus opening new pathways for controlling reaction rates and potentially tuning reaction selectivity.

## 2 Results and Discussion

We synthesized ITO NCs by a slow-injection method, adding a metal precursor solution dropwise to a hot oleyl alcohol solution.^27,44^ Using these synthesized ITO NCs, we prepared compact NC assemblies by templating monolayers at the liquid–air interface, producing a NC film atop the diethylene glycol (DEG) subphase. We then introduced a 4-azidobenzoic acid (ABzA) solution in the DEG phase, enabling ligand exchange to replace the native oleate ligands on the NC surface with ABzA. This molecule features an azide functional group that serves as the reaction center and a carboxylate group, which ensures chemisorption onto the NC surface. Based on the intensity change of the C–H stretching vibration in Fourier-transform infrared (FT-IR) spectroscopy, ligand exchange results in approximately 40% of the native oleate ligands on the NCs being replaced with ABzA molecules (Figure S1). As seen in scanning electron microscopy (SEM) images of ITO NC monolayers with ABzA molecules on the surface (Figures 1a and S3), the ITO NCs assembled into compact arrays regardless of their size or doping concentration. The ABzA vibrational modes are readily observed by FT-IR of the ligand-exchanged ITO NC monolayers, especially the asymmetric stretching mode of the azide group located at 2126 cm^–1^, as reported in our previous systematic study of SEIRA in similar samples.^41^ However, we did not observe any peak splitting of the vibrational mode, confirming that the system remains in the weak coupling regime.

**Figure 1:**
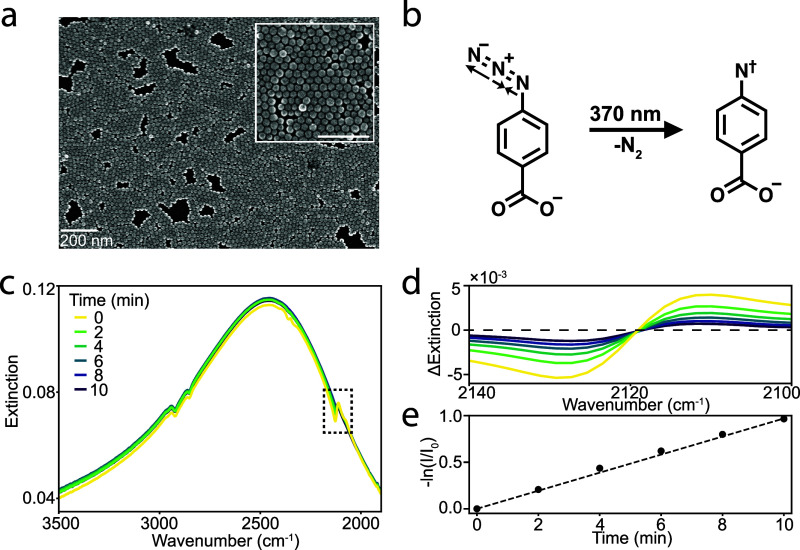
ITO NC monolayer used for kinetic control over 4-azidobenzoic acid (ABzA) photolysis. (a) Top-down SEM image of a 0.9% Sn-doped ITO NC monolayer, where native oleate ligands have been exchanged with ABzA molecules. The inset shows the NC film at high magnification, with the scale bar indicating 200 nm. (b) Photolysis of ABzA following illumination with 370 nm light results in a loss of N_2_ and forms a reactive intermediate. Arrows on the reactant molecule highlight the asymmetric stretching mode of the azide group, which is lost after the reaction proceeds and is monitored to quantify reaction progress. (c) Time evolution of the extinction spectra of a 2% Sn-doped ITO NC monolayer under illumination from a 370 nm light-emitting diode (LED), highlighting the asymmetric azide stretching mode. (d) The asymmetric (Fano line shape) azide stretching mode decays as a function of UV illumination time after subtraction of the CPR background using polynomial fitting. We recorded the difference between the maximum and minimum of the vibrational signal at each time point. (e) The vibration intensity was normalized to its initial value, and the negative logarithm was taken, resulting in a linear progression as a function of time, indicating a first-order kinetic process.

Upon illumination with UV light (370 nm from an LED, at 13.3 mW cm^–2^), we observed the degradation of the azide-functionalized molecule on the NC surface due to N_2_ loss from the azide group (Figure 1b). The reaction did not proceed spontaneously without UV illumination (Figure S4). The reaction rate was tracked using the SEIRA-enhanced vibrational signature of the azide group, enabled by coupling to the ITO NC CPR. Specifically, we monitored the intensity of the asymmetric stretching located at 2126 cm^–1^. To verify the utility of this SEIRA-enhanced mode for quantifying degradation of the azido reactant, we compared the rate of intensity loss to that of the symmetric stretching mode of the azide group at 1286 cm^–1^, which exhibited indistinguishable kinetics, indicating that the SEIRA-enhanced vibrational signal accurately reflects the actual photolysis rate of azide degradation (Figure S5 and Table S1).

To quantify the photolysis kinetics, we collected spectra as the reaction progressed, observing that the CPR frequency and intensity remain relatively constant, while the vibrational signal diminishes (Figure 1c). For analysis, we subtracted the CPR background at each time point using polynomial fitting, obtaining vibrational spectra of the azide asymmetric stretch that exhibits a Fano line shape (Figure 1d). We defined the vibrational intensity as the difference between the peak and the dip in the Fano resonance. We then normalized the vibrational intensity by dividing it by the initial value (at 0 min of UV exposure) and took the negative natural logarithm to reflect the changing abundance of azide groups in the probed volume, which results in a well-defined linear trend as a function of time (Figure 1e). This linearity indicates that the photodegradation follows first-order kinetics over this time period, and the rate constant (*k*) is obtained from the slope of the graph. Throughout UV illumination of the sample, we also monitored the C–H vibration (2900 cm^–1^) of the residual native oleic acid ligand for changes indicating nonspecific ligand degradation, where we found no change in the signal whatsoever (Figure S2).

To assess whether CPR-vibrational coupling might influence *k*, we first compared the photolysis rates of ITO NC monolayers with varying particle diameters (Figures 2a, S6, and Table S2). Uniformly sized ITO NCs ranging from 11 to 26 nm in diameter were used as building blocks for the metasurfaces while maintaining a nearly constant CPR frequency (ω_CPR_) of around 2750 cm^–1^ (Figure 2a). Accordingly, we maintained a constant detuning (Δω = ω_CPR_ – ω_vib_), defined as the frequency difference between the CPR and the molecular vibration, while varying the NC size in the monolayer assemblies. Based on our previous work, the number of molecules coupled to the CPR and the interparticle spacing are approximately constant as the NC size is varied in this range.^41^ But, the CPR resonance and the coupling between the CPR and the vibrational mode grow stronger as the NC size increases.^41^

**Figure 2:**
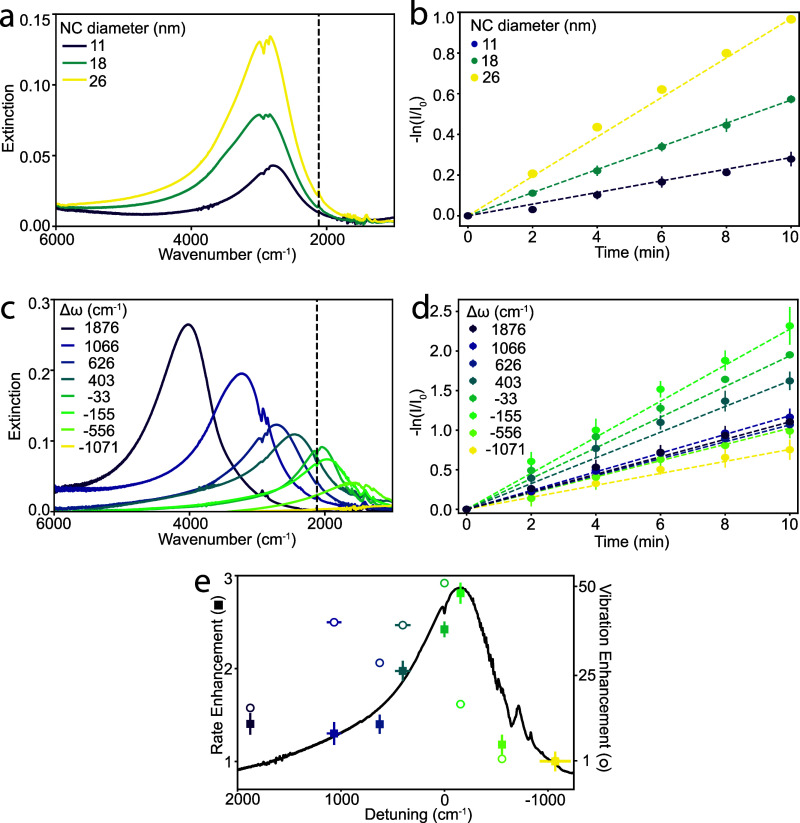
Photolysis rate variation as a function of NC size and frequency detuning. (a) Extinction spectra of different-sized ITO NC monolayers with similar frequency detuning (Δω ≈ 750 cm^–1^). The dashed line indicates the frequency of the asymmetric stretching mode of the azide group. (b) Kinetics of azide photolysis tracked through the decay of the vibrational mode in ITO NC assemblies with varying NC diameters. Dotted lines indicate linear fits used to extract the photolysis rates. Error bars represent standard deviations from three replicates for each NC monolayer. (c) Extinction spectra of ITO NC monolayers with similar diameters (27.5 ± 1 nm) but different Δω values, controlled by the Sn concentration in the ITO NCs. Sn concentrations are listed in Table S3. (d) Kinetics of azide photolysis for NC monolayers with different Δω values are shown in (c). (e) Rate enhancement (filled squares, relative to undoped In_2_O_3_) as a function of detuning, showing a trend that follows the extinction spectrum of the NC monolayer for which the photolysis rate is greatest (Δω = −155 cm^–1^). Open circles indicate the vibration signal enhancement factor from the asymmetric azide stretching mode (ω_vib_). We calculate this enhancement factor by dividing the vibrational signal by the azide vibrational signal on the undoped In_2_O_3_ (Δω = −1071 cm^–1^) NC monolayer.

We found that *k* increased from 0.028 (±0.003) to 0.099 (±0.003) min^–1^ as the NC diameter increased from 11 to 26 nm (Figure 2b). This trend was reproduced across three independent trials, showing consistent photolysis rates (Figures 2b and S7). Monolayers of larger NCs exhibit greater polarization under plasmon excitation, as evidenced by a higher extinction peak intensity (Figure 2a), which contributes to a stronger antenna effect that concentrates the optical near-field in the inter-NC spaces. In addition, consistent with our previous work,^41^ temporal coupled mode theory (TCMT) analysis reveals that the near-field coupling strength between the CPR and molecular vibrations increases with NC size (Figure S8). These two combined effects enhance the electric field experienced by molecular vibrations, resulting in a greater vibrational signal intensity for larger NCs. The results suggest that this stronger near-field coupling at the vibrational frequency is responsible for the accelerated photolysis observed for larger NCs (Figure 2b).

To better understand how coupling to molecular vibrations impacts the photolysis rate, we next fixed the NC diameter (27.5 ± 1 nm) and varied the Sn doping concentration. Sn doping from 0% to 7% tunes the CPR frequency across the IR region (Figures 2c, S6, and Table S3), enabling a study of the effect of Δω on *k*. Instead of referring to Sn doping concentrations, we use Δω to highlight the effect of CPR frequency on the photolysis reaction throughout the manuscript (doping concentrations for each sample are provided in Table S3). Regardless of the variety of azide vibrational lineshapes seen with different Δω, photolysis was observed to diminish the vibrational signal for NC surface-bound ABzA molecules (Figure S9). However, the photolysis rate increased as Δω shifted from the most negative value (lightly doped NCs) toward smaller |Δω|, with *k* rising from 0.082 (±0.006) min^–1^ at Δω = −1070 cm^–1^ to a maximum of 0.23 (±0.02) min^–1^ at Δω = −155 cm^–1^ (Figure 2d). Beyond this point, Δω increases and *k* gradually decreases to 0.12 (±0.01) min^–1^. The falloff in the photolysis rate away from Δω = 0 cm^–1^ occurs despite the CPR becoming monotonically more intense at higher Δω and despite the CPR passing through resonance with other vibrational modes of ABzA at both lower and higher ω. These results indicate that near-zero Δω facilitates photolysis, providing evidence that strengthening the CPR-vibrational coupling enhances reaction kinetics.

To further investigate whether CPR-vibrational coupling may be governing the variations in *k*, we sought to compare the Δω dependence of the reaction rate to the CPR spectral line shape. In previous studies of vibrational strong coupling effects on reaction rates, the dependence of kinetic changes on detuning was observed to resemble the line shape of the corresponding molecular vibrational mode.^1,2^ In our case, the CPR is broad, contrasting with the narrow line widths of Fabry-Pérot microcavity modes exploited in those earlier investigations. To compare the measured enhancement of the photolysis rate with the CPR spectrum, we overlaid the normalized *k* at each Δω with the CPR extinction spectrum for Δω = −155 cm^–1^, which shows the most significant photolysis rate (Figure 2e). The photolysis rate was normalized to the case of Δω = −1071 cm^–1^ (*k*_In2O3_), corresponding to In_2_O_3_ NCs with weak plasmonic character, to highlight the relative rate enhancement. The general correspondence between the action spectrum and the CPR line shape reinforces that the rate enhancement is a resonance effect that follows closely the spectral overlap between the CPR and the azido vibrational stretching mode. This mode lies along the reaction coordinate, leading to N_2_ dissociation, suggesting that even in the weak coupling regime, mode-specific plasmon-vibrational coupling can modulate the progress of chemical reactions.

To assess whether the rate enhancement trend is a product of the frequency-dependent near-field intensity, we quantified the vibrational signal enhancement as a function of the detuning (Figure 2e). We measured vibrational enhancement, which serves as a probe of near-field intensity, by normalizing the vibrational signal to the control sample, resulting in a trend that also mimics the CPR line shape, but is shifted in detuning.^41^ The offset in Δω between the maximum photolysis rate and the vibration signal enhancement suggests a more direct role of the modal resonance in driving the observed rate enhancement than that of the SEIRA effect.

Because the photolysis reaction occurs on the surface of the NCs, it is possible that UV light absorption by the ITO NCs and the resulting photogenerated charge carriers could influence the observed UV photolysis rates. To rule out this direct photoexcitation effect, we measured the UV–visible absorption spectra of ABzA-decorated ITO NC monolayers on UV-transparent sapphire substrates (Figure S11), focusing on absorption at the excitation wavelength of 370 nm, which is below, but close to, the optical band gap of ITO. After converting the spectra into Tauc plots, we found that the band gap of the ITO NC assemblies, regardless of doping concentration, shows minimal absorption at the photon energy of the LED used for photolysis (Figure 3a). Although sub-bandgap absorption via defect states is possible, the UV–vis spectral trend shows a monotonic decrease in absorption at 370 nm with increasing Δω, since higher Sn doping concentrations expand the optical band gap.^45^ This trend does not correlate with the nonmonotonic behavior of the rate constant *k* as a function of Δω (Figure 2d,e). Therefore, it is unlikely that photoexcitation of the ITO NCs accounts for the enhanced *k* values observed at specific Δω values. Additionally, we measured the absorption spectra of the ABzA molecules before and after ligand exchange. The spectra reveal that NC-bound molecules exhibit broader absorption extending into the visible region, including increased absorption at 370 nm, compared to the free molecules in solution (Figure S12). These observations support the assessment that photolysis proceeds following UV excitation of NC-bound ABzA and that the reaction rate is enhanced by vibrational coupling with the ITO NCs.

**Figure 3:**
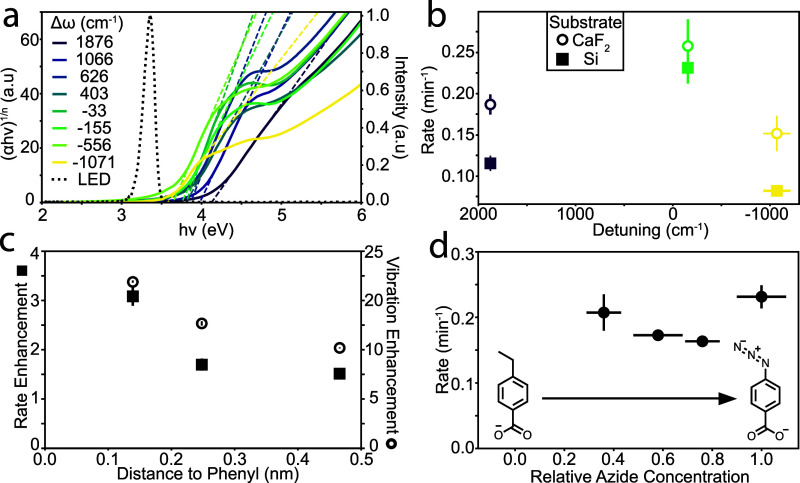
Control experiments to demonstrate that the CPR-vibration resonance drives the enhanced photolysis rate. (a) Tauc plots derived from UV–vis extinction spectra of ITO NC monolayers, showing a monotonically increasing band gap at higher Sn doping concentration. The LED emission profile is overlaid as a dotted black line. Optical band gaps (Table S4) were determined by extrapolating the linear region of the curve to the *x*-axis (dotted lines). (b) Differences in reaction rates for ITO NCs with varying Δω, for assemblies deposited on Si (filled squares, select data points from Figure 2e) and CaF_2_ (open circles) substrates. (c) Vibrational intensity (open circles) and rate enhancement (closed squares) relative to the same molecule on In_2_O_3_ NCs as a function of the length of the molecule from the NC surface to the phenyl ring. (d) Reaction rates in NC monolayers with Δω = −155 cm^–1^ at various ABzA concentrations on the NC surface. A mixture of ABzA and 4-ethylbenzoic acid was used to control the relative surface density of the azide functionalized molecules.

To rule out the possibility that substrate heating from the 370 nm LED somehow underpins the observed resonant enhancement, we replaced the UV-absorbing Si substrate with CaF_2_, which is transparent from the UV to the mid-IR range (Figure S13). The trend of increased photolysis rate at small absolute Δω values remained evident (Figure 3b), confirming that the trend is not related to optical heating. Notably, *k* was approximately 20% higher on CaF_2_ than on Si for Δω = −133 cm^–1^. Since TCMT (Figure S14) predicts similar coupling strengths across both substrates, we attribute the higher *k* on CaF_2_ to increased NC polarization at IR frequencies, reflected in greater extinction (Figure S14). This stronger polarization, which we observe even in weakly plasmonic In_2_O_3_ NCs, likely contributes to the modest increase in photolysis rate on CaF_2_, where a stronger vibrational signal is observed from ABzA bound to 0% Sn-doped ITO NCs on a CaF_2_ substrate than on Si (Figure S14).

To evaluate the generality of resonant enhancement on the photolysis reaction, we varied the ligand structure from ABzA, where the azide group is directly attached to the aromatic ring bearing the carboxylate, to ligands containing an azidophenyl group linked via an aliphatic chain to a terminal carboxylic acid. Specifically, we compared the vibrational intensity and photolysis kinetics of (4-azidophenyl)­acetic acid (C1 ABzA) and 4-(4-azidophenyl)­butyric acid (C3 ABzA) with those of ABzA. Despite the longer aliphatic chains, we still observed a persistent rate enhancement with on-resonant ITO NCs (Δω ≈ −130 cm^–1^, Figures S15 and S16) compared to the off-resonant case (Δω ≈ −1000 cm^–1^), similar to the trend seen with ABzA. However, when comparing the rate constant ratios between these two conditions (Figure 3c), we observed a smaller magnitude of enhancement than found for ABzA, and a corresponding lower vibrational intensity. We suspect that this weakened enhancement arises from a reduction in electric field intensity as the aliphatic chain length increases. The residual native oleate ligands prevent a precise templating of the interparticle distance based solely on ligand length, and we were unable to detect significant differences in interparticle spacing by grazing incidence small-angle X-ray scattering and Fourier-transformed SEM images (Figure S17). Nevertheless, we expect longer ligands to change the distance of the azide groups from the NC surface within the gaps, thus changing the strength of the interaction between the molecule and the CPR mode.

The retention of a resonant enhancement trend in rate constant ratios, even with the addition of an aliphatic spacer in the azido reactant molecules, helps rule out a significant contribution from photogenerated charge carriers produced by ITO NC band gap absorption. Charge transfer rates decrease exponentially with distance, following the relation *e* ^–β*x*^, where β is the distance decay constant, and *x* is the separation between the NC surface and the functional group. For aliphatic chains, β is approximately 9 nm^–1^,^46^ while for phenyl groups it is around 4 nm^–1^,^47^ due to greater electron delocalization. Therefore, we define *x* as the distance between the carboxylate group and the carbon atom directly bound to the phenyl ring, since the phenyl unit can more effectively mediate electron transfer than an aliphatic chain. From ABzA to C3 ABzA, this increasing length should reduce the charge transfer rate by approximately 92%. In contrast, the observed photolysis rate enhancement, calculated by comparing the Δω ≈ −130 cm^–1^ and Δω ≈ −1000 cm^–1^ cases (with In_2_O_3_ NCs), only decreases by about 45%. Furthermore, using time-dependent density functional theory (TDDFT) at the B3LYP level, we calculated the lowest unoccupied molecular orbital of ABzA to be −1.96 eV. This level is significantly higher than the expected Fermi energy of ITO NCs (−4.8 eV, regardless of doping level),^48^ supporting our assessment that the reaction enhancement does not arise from spontaneous charge transfer. These results collectively support the conclusion that the observed photolysis enhancement is driven by near-field interactions, i.e., CPR-vibrational coupling, rather than a charge transfer mechanism.

Since the enhancement relies on near-field coupling between the molecules and CPR, the behavior of the photolysis reaction may differ from previous studies of reactions under collective vibrational strong coupling, particularly those in which reactivity was strongly influenced by the concentration of molecules coupled to the photonic structure.^49^ To examine the effect of reactant concentration on CPR–vibrational coupling and photolysis kinetics, we introduced a nonphotoactive ligand, 4-ethylbenzoic acid, whose structure is similar to ABzA (Figures 3d and S18). By diluting the photoactive ligand with the nonphotoactive ligand at various ratios, we systematically varied the number of azide-functional ligands within the NC gap. The extent of azide dilution was quantified by measuring the intensity of the azide asymmetric stretch, normalized to that of similar NC monolayers that were functionalized with pure ABzA. Regardless of the dilution factor, the photolysis rate constant remained nearly unchanged and showed no trend with dilution, which stands in contrast with prior studies that reported strong concentration dependence of reactivity under vibrational strong coupling.^49^ While further investigation is warranted, we suspect that the absence of collective behavior (*e.g.*, lack of Rabi splitting that depends on increasing molecular population) may result from the lossy nature of our plasmonic “nanocavity,” which prevents coherent energy exchange between the electromagnetic field and the molecular ensemble. We therefore conclude that photolysis in this system occurs under weak near-field coupling conditions, where a consistent rate enhancement is observed, regardless of molecular surface coverage.

To understand how CPR coupling can enhance the reaction rate, we first characterized the relevant excited states using TDDFT (Figure S19). Analysis of oscillator strengths (section 12 and Table S5) identifies S_2_ as the photoactive state within the experimental excitation window. Prior studies on phenyl azides^50,51^ propose a mechanism in which photoexcitation to S_2_ is followed by internal conversion (IC) to S_1_ (mediated by vibrational excitation on the S_2_ surface and a Landau–Zener type nonadiabatic transition to S_1_, see Figure S21), and subsequent dissociation on the S_1_ PES. On this basis, we hypothesize that the CPR facilitates the vibrational excitation on the S_2_ surface, thereby facilitating IC and accelerating the reaction.

To test this hypothesis, we computed the N_3_ asymmetric-stretch frequencies in the ground and S_2_ states (2270 and 2103 cm^–1^, respectively), yielding a vibrational mode red shift of around −167 cm^–1^ (Figure 4a). This red shift in the excited state explains why the maximum rate enhancement occurs at slightly negative detuning (Δω = −155 cm^–1^) relative to the ground-state vibrational frequency (Figure 2d). Because the plasmon–cavity quality factor, defined as ω_CPR_/Δω_CPR_, is low in our system (*Q* ≈ 2.3), the CPR mode is expected to effectively serve as an energy source^17,52^ for vibrational excitation (Figure 4b), which has been demonstrated by our previous work using exact quantum dynamics simulations and analytic rate constant theory. To quantify the resulting *net enhancement* rate of vibrational excitation due to the CPR, defined as *k*_VSC_ = *k* – *k*_0_, we use a rate theory based on Fermi’s Golden Rule (FGR, Section S13 of the Supporting Information)­

$$k_{\mathrm{VSC}}=2\alpha \langle \cos^{2}\;\theta \rangle \int_{0}^{\infty }d\omega \times J_{\mathrm{eff}}(\omega )A(\omega -\omega_{0})\times n(\omega )$$ 1

Here, with ⟨cos^2^θ⟩ = 1/3 as the isotropic dipole angle orientation relative to the field polarization direction, $A\left(\omega -\omega_{0}\right)$ is the broadening function of the excited state molecular vibrations, where ω_0_ is estimated from the vibrational normal-mode frequency in the electronic excited state, *n*(ω) = [exp­(*ℏ*ω/*k*_B_ *T*) – 1]^−1^ is the Bose–Einstein distribution for the photonic mode with frequency ω. The effect of the cavity mode and its environment on the system is captured by the effective spectral density:

$$J_{\mathrm{eff}}\left(\omega \right)=\frac{2g_{c}^{2}\left(\omega_{c}\right)\tau_{c}^{-1}\omega_{c}\omega }{{\left(\omega_{c}^{2}-\omega^{2}\right)}^{2}+\tau_{c}^{-2}\omega^{2}}$$ 2

with τ_c_ as the plasmon-mode lifetime (related to the quality factor as $Q=\omega_{c}\tau_{c}$ ) and *g*_c_ (ω_c_) = η_c_ω_c_ ·|μ_S_0__| is the cavity frequency-dependent light-matter coupling with the ground state vibrational transition intensity, which is directly measurable from the vibrational excitation in the electronic ground state. *J*_eff_(ω) is in good agreement with the experimental extinction spectrum (Figure 4b). Finally, the parameter α = |μ_S_2__|^2^/|μ_S_0__|^2^ ≈ 0.3 includes the ratio of the vibrational transition intensity for the vibrations in the electronic excited state |S_2_⟩ vs the electronic ground state |S_0_⟩.

**Figure 4:**
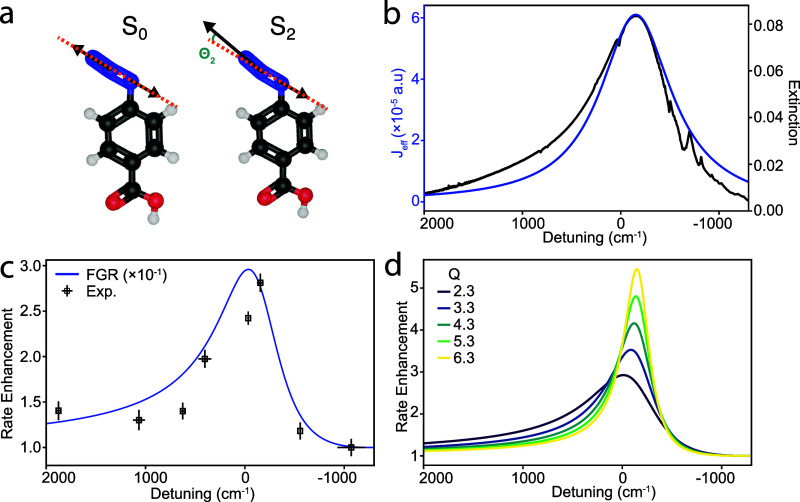
Resonant vibrational excitation-based theoretical model to predict the CPR-enhanced frequency-dependent photolysis. (a) Atomic displacements (arrows) of the relevant normal mode of the azide functional group at the S_0_ and S_2_ states of the ABzA molecule. The orange dashed line serves as a visual guide to show the angular difference in the vibrational angle between the two states, Θ_2_. (b) Effective spectral density *J*_eff_(ω) (blue) and experimental extinction spectrum of the NC monolayer with Δω = −155 cm^–1^ (black). (c) Experimental (black squares) and FGR (blue) rate enhancements as a function of detuning. All calculations are done with a plasmon-cavity quality factor *Q* = 2.3. (d) Theoretical predictions (eq 1) of quality factor-dependent rate enhancement, up to 5 times larger with Q=6.3 compared to Q=2.3 , and a peak-position gradually red-shifted. The values have been rescaled by a factor of 10 as in (c).

Using eq 1, we evaluate *k*_VSC_ as a function of the cavity frequency (ω_*c*_) with a plasmon–cavity quality factor *Q* = ω_*c*_τ_c_ ≈ 2.3 (corresponding to a cavity lifetime τ_c_ ≈ 6.2 fs). The coupling strength *g*_c_(ω_c_), which depends on the Sn doping concentration in ITO NCs through stronger near-field enhancement at higher doping levels, was interpolated from Figure S10 using the doping-concentration-dependent TCMT analysis of our experimental vibrational spectra (Figure 2c). The vibrational rate enhancement predicted by FGR reproduces the experimentally observed trend in the reaction rate enhancement (Figure 4c). More importantly, it captures the fundamental physics that the rate enhancement (the “action spectrum”) closely follows the effective spectral density of the plasmonic mode. Note that the FGR rate is approximately one order of magnitude larger than the experimentally measured value (Figure 4c). Such overestimation is commonly encountered in second-order perturbation theory and FGR.^17,51^ Another source of error is the assumption in the FGR formulation that all vibrational excitations contribute to the rate enhancement. In the present system, however, non-radiative relaxation can effectively reduce the rate constant (Figure S21).^52^ Nevertheless, the resulting discrepancy remains within the validity and expected accuracy of perturbative FGR treatments.^53,54^ This result indicates that the resonant CPR efficiently activates the asymmetric stretching of the N_3_ mode from the S_2_ state, facilitating the reaction dynamics. Therefore, the CPR resonates with the vibrational frequency, enabling efficient N–N bond cleavage and the loss of the azide group.

We can leverage this model to predict the kinetic enhancement based on the carbon chain length. Since we observed a slight decrease in the *g* value as the carbon length increases, obtained from TCMT modeling, we interpolated the *g* trend as a function of the carbon chain length (Figure S22). Using a given *g* value, we can qualitatively reproduce the rate enhancement trend shown in Figure 3c with our FGR, although the decrease in rate enhancement with longer carbon chains is less dramatic compared to that in the experiments (Figure S22). We suspect that this discrepancy arises from the simplified treatment of the reaction process in the FGR approximation.

Finally, with the given model, we can design a strategy to enhance the photolysis rate further. By varying the quality factor, where Δω_CPR_ is the full width at half-maximum of the CPR line shape, we expect the photolysis rate enhancement to increase from 2.9 to 5.0 as the quality factor rises from 2.3 to 6.3 (Figure 4d). This result suggests that even greater enhancements are achievable. A practical route to this strategy is substituting ITO NCs with compositions exhibiting higher electron mobility, such as cerium-doped indium oxide (56 cm^2^/V·s)^55^ or indium-doped cadmium oxide (70 cm^2^/V·s).^56^ Indeed, our previous work demonstrated that indium-doped cadmium oxide NC assemblies exhibit a narrower line width than ITO NC assemblies.^57^ With such experimental refinements, a further enhancement in reaction rates may be realized.

## 3 Conclusion

Our study demonstrates that the CPR of ITO NC monolayers can couple to the vibrational modes of surface-bound ABzA molecules, thereby accelerating UV-driven photolysis. We show that larger NCs, which generate stronger electric fields, and resonance conditions achieved through optimal Sn doping contribute to an enhanced photolysis rate. We rule out alternative explanations such as UV absorption by ITO or the substrate and demonstrate that this resonant enhancement effect is robust across varying ligand lengths and molecular concentrations. Finally, we develop a theoretical model indicating that CPR–vibration coupling facilitates crossing of the excited-state energy barrier, thereby promoting bond dissociation.

Building on this work, we propose that NC-based metamaterials could bridge the gap between weak and strong vibrational coupling regimes. Looking forward, NC assemblies can be readily integrated with photonic structures,^58^ providing more degrees of freedom in the design of optical modes and vibrational coupling conditions than NCs alone. This integration would allow systematic investigation of quality factor-dependent reaction rates, as previously predicted in theoretical studies,^52^ and guide the design of hierarchical structures optimized for reaction modulation. Moreover, the CPR frequency of our system is tunable, offering potential for product-selective control by matching the resonance to specific vibrational modes of reactants along the desired reaction coordinate, analogous to the envisioned opportunities for vibrational-mode-selective excitation with IR light pulses.^59^ The potential utility of this strategy extends beyond photolysis to other applications, including ionic conductivity tuning,^60^ modifying intramolecular vibrational-energy redistribution to affect chemical reactivity,^61^ and more.

## 4 Experimental Section

### 4.1 Materials

All chemicals were used as received without any additional purification. Indium­(III) acetate [In­(ac)_3_, 99.99%, Sigma-Aldrich], tin­(IV) acetate [Sn­(ac)_4_, Sigma-Aldrich], oleic acid (OA, 90%, technical grade, Sigma-Aldrich), oleyl alcohol (OLA, 90%, technical grade, Sigma-Aldrich), and octadecene (ODE, 85%, technical grade) were used to synthesize the NCs. Hexane (≥99.9%, Fischer Scientific), diethylene glycol (DEG, 99%, Sigma-Aldrich), and acetonitrile (99%, Fischer Scientific) were used for the assembly of NC monolayers. 4-Azidobenzoic acid (ABzA, TCI America, ≥97%), (4-azidophenyl)­acetic acid (C1 ABzA, Life Chemicals Inc.), 4-(4-azidophenyl)­butyric acid (C3 ABzA, 98%, Chem-Impex International Inc.), and 4-ethylbenzoic acid (99%, Sigma-Aldrich) were used for exchanging ligands on the NC surfaces.

### 4.2 Synthesis of ITO NCs

NCs were synthesized by a slow-injection method based on previous reports.^27,44^ For ITO NCs around 27 nm, we dissolved 8 × (1 – *x*) mmol of In­(ac)_3_ and 8 × *x* mmol of Sn­(ac)_4_ in 16 mL of OA and 8 mL of ODE. The value of *x* corresponds to the desired doping concentration. The solution was heated to 150 °C under a N_2_ atmosphere for 2 h. In a separate flask, 13 mL of OLA was degassed under vacuum for 1 h at 110 °C and then heated to 290 °C under nitrogen flow. We injected 21 mL of the precursor solution into the OLA at a rate of 0.3 mL min^–1^ using a syringe pump. After the injection was complete, the reaction mixture was maintained for an additional 20 min and then allowed to cool naturally to room temperature. The product was washed three times with ethanol and redispersed in 10 mL of hexane for storage.

For 11 and 18 nm ITO NCs, we dissolved 0.5 × 0.98 mol L^–1^ of In­(ac)_3_ and 0.5 × 0.02 mol L^–1^ of Sn­(ac)_4_ in OA and degassed the solution under vacuum for 1 h at 100 °C. The precursor solution was then heated to 150 °C for 2 h under nitrogen. Separately, 13 mL of OLA was heated under nitrogen to 290 °C, followed by slow injection of 25 mL of the precursor solution at a rate of 0.35 mL min^–1^ using a syringe pump. The injected volumes were 4 and 16 mL of precursor for the 11 and 18 nm ITO NCs, respectively. The OLA injection step was repeated after an additional 10 mL of metal precursor was added. The synthesized NCs were centrifuged with excess ethanol after dilution with hexane, and the washed NCs were finally redispersed in hexane.

### 4.3 ITO NC Characterization

ITO NC monolayers were imaged using a Hitachi S5500 scanning transmission electron microscope in secondary electron mode. For inductively coupled plasma optical emission spectroscopy (ICP-OES), the ITO NC solution was first dried and digested with aqua regia. After 2 days of digestion, the solution was diluted to 2% nitric acid and analyzed using an Agilent 7500ce spectrometer to determine the elemental concentrations. The sizes of the synthesized ITO NCs were analyzed by small-angle X-ray scattering (SAXS) using an in-house SAXSLab Ganesha instrument. We also utilized the same instrument for grazing-incidence small-angle X-ray scattering (GISAXS) to measure the interparticle distance of the NC monolayers.

### 4.4 ITO NC Monolayer Formation

A double-sided-polished silicon wafer was placed in a Teflon trough containing 5 mL of DEG. Then, 10–30 μL of ITO NC solution in hexane (3 mg mL^–1^) was drop-cast onto the DEG subphase to form a monolayer of ITO NCs. For ligand exchange, a 10 mM ligand molecule in acetonitrile solution was prepared and injected into the DEG subphase after monolayer formation, followed by a 30-min waiting period. The substrate was subsequently washed with pure acetonitrile to remove any excess molecules physisorbed on the NCs. For other substrates, the silicon wafer was simply replaced with the desired substrate, and the same procedure was followed. For the mixed-ligand surface shown in Figure 3d, the relative azide concentration was obtained by dividing the vibrational signal at 0 min by the vibrational signal from the case with only ABzA on the NC surface without any 4-ethylbenzoic acid.

### 4.5 Photolysis and Optical Measurements

For photolysis, the silicon substrate was placed next to the LED lamp (Kessil PRL-370 Gen 2) and irradiated in 2 min increments. The extinction spectra of the NCs on double-sided-polished Si wafers were then measured using a Bruker Vertex 70 FT-IR spectrometer in transmission mode with a 0.5 cm beam diameter holder. This process was repeated for 10 min to determine the photolysis rate. We used an Agilent Cary 5000 instrument to obtain the UV–vis absorption spectra of NC films on sapphire substrates.

## Supplementary Material



## Data Availability

The data supporting the findings of the manuscript are available upon a reasonable request from the corresponding authors.

## Notes

Colloidal ITO NCs and their film properties, measurement kinetics, UV–vis absorption of the NC films, temporal coupled-mode theory fitting results, and theoretical framework (PDF)
