# Supplementary material for: Resonant Enhancement of Photolysis through Plasmon-Vibrational Coupling
Source: J Am Chem Soc. 2026 Jan 22;148(4):4670–9. doi: 10.1021/jacs.5c20841 (PMC12879941; doi:10.1021/jacs.5c20841)
Supplement: Supplementary file 1 [file ja5c20841_si_001.pdf]

Supplementary Information: Resonant enhancement of photolysis through plasmon-vibrational coupling

Woo Je Chang,[†,‡] Logan J. Carr,[¶,‡] Sebastian Montillo Vega,[§] Priyansh Vora,[†] Pengfei Huo,[*,§] and Delia J. Milliron[*,∥,†,¶]

†*McKetta Department of Chemical Engineering, University of Texas at Austin, Austin, Texas 78712, United States*

‡*These authors contributed equally to this work*

¶*Department of Chemistry, University of Texas at Austin, Austin, Texas 78712, United States*

§*Department of Chemistry, University of Rochester, Rochester, New York 14627, United States*

∥*Department of Chemical Engineering, University of Michigan, Ann Arbor, Michigan 48109, United States*

E-mail: pengfei.huo@rochester.edu; milliron@umich.edu

1 Ligand Exchange

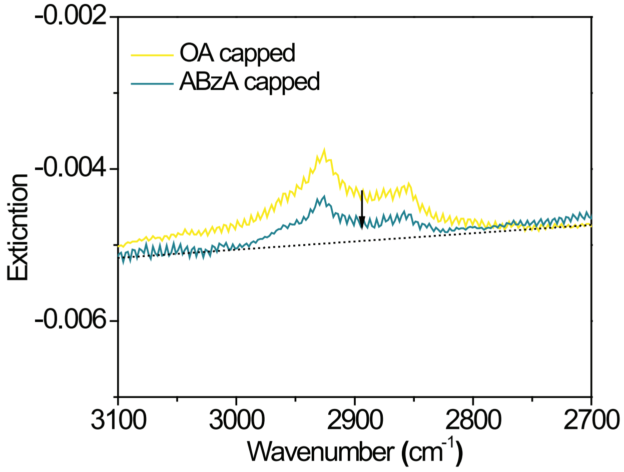

Figure S1: **Ligand exchange efficiency based on oleate peak removal.** We evaluate the C–H vibrational mode around 2900 cm^{-1}, where approximately 40% of the signal disappears upon ABzA ligand exchange.

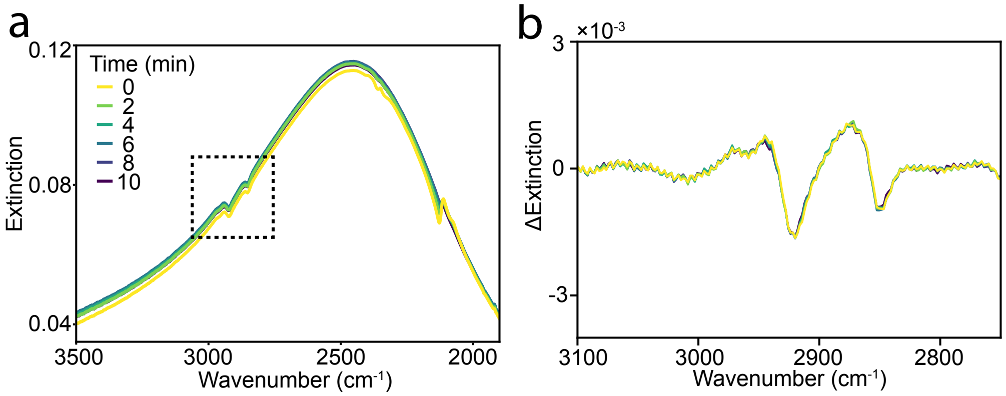

Figure S2: **Native oleic acid ligand signal under UV illumination.** (a) Illumination time-dependent extinction spectra of post-ligand exchange ITO NC monolayer (same as Figure 1c), with a box surrounding the C-H vibrational mode. (b) C-H vibration region is fit with a polynomial to subtract out the CPR background, where the oleic acid signal shows no change throughout UV illumination time.

2 ITO NC Monolayer SEM Images

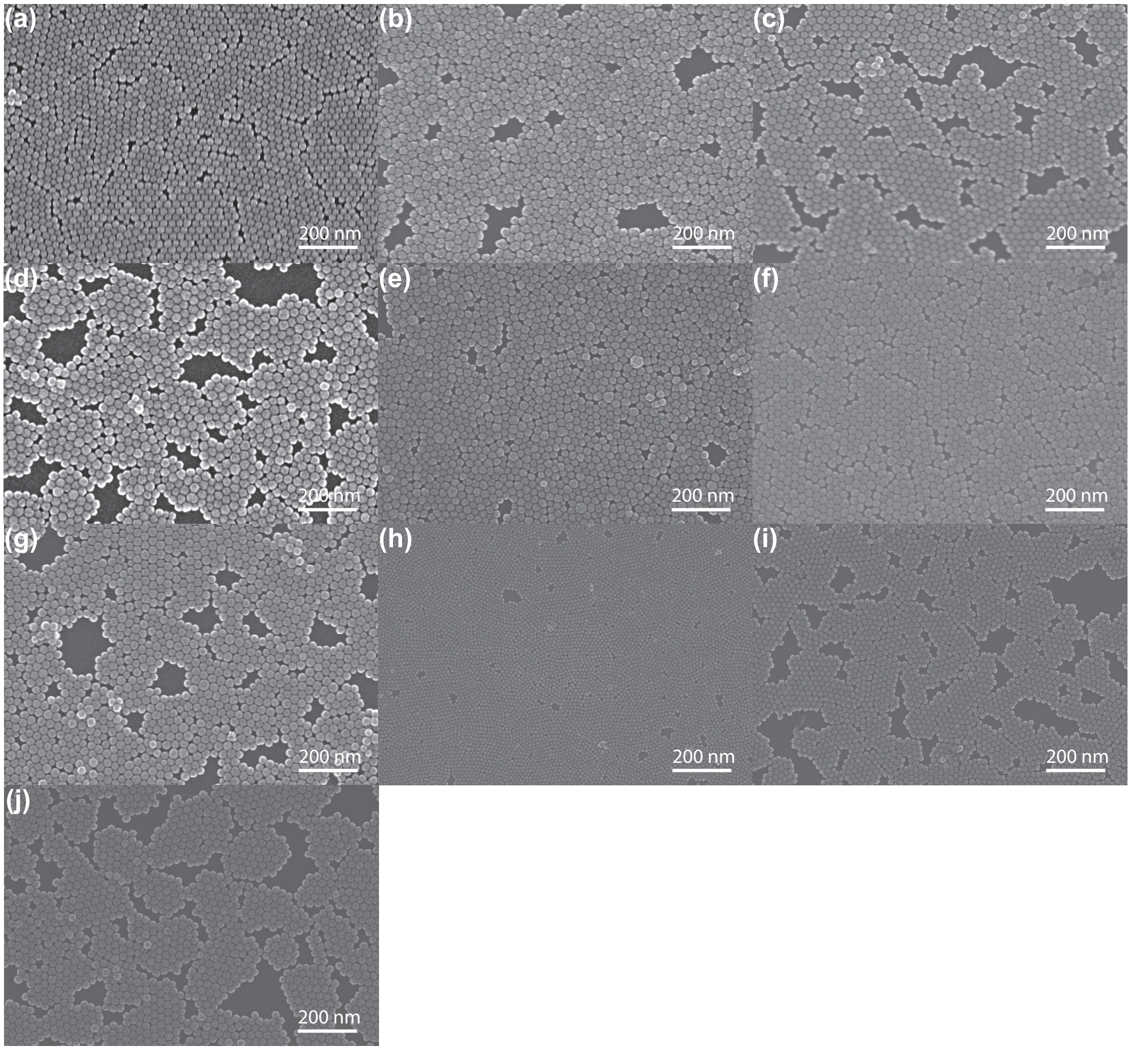

Figure S3: **Scanning electron microscope (SEM) images of ITO NC monolayers with various doping and sizes.** (a) 0% Sn and 27.4 nm, (b) 0.5% Sn and 27.0 nm, (c) 1% Sn and 29.4 nm, (d) 2% Sn and 27.4 nm, (e) 2.3% Sn and 27.0 nm, (f) 3.3% Sn and 26.2 nm, (g) 7.0% Sn and 27.8 nm, (h) 2.1% Sn and 11.2 nm, (i) 2.4% Sn and 18.4 nm, and (j) 2.7% Sn and 25.6 nm ITO NCs monolayers.

3 Light Dependency for Reaction

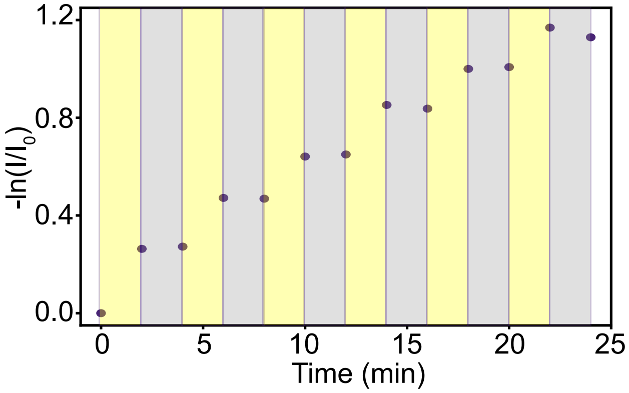

Figure S4: **The asymmetric vibrational mode decays under repeated light on/off illumination for 7% Sn doped ($\Delta\omega = 1899$ cm^{-1}) ITO NC monolayers with ABzA molecules.** We observed degradation of the azide asymmetric stretching mode only during LED illumination (yellow boxes), whereas no degradation occurred in the absence of light (dark boxes).

4 Frequency Dependent Degradation Rate

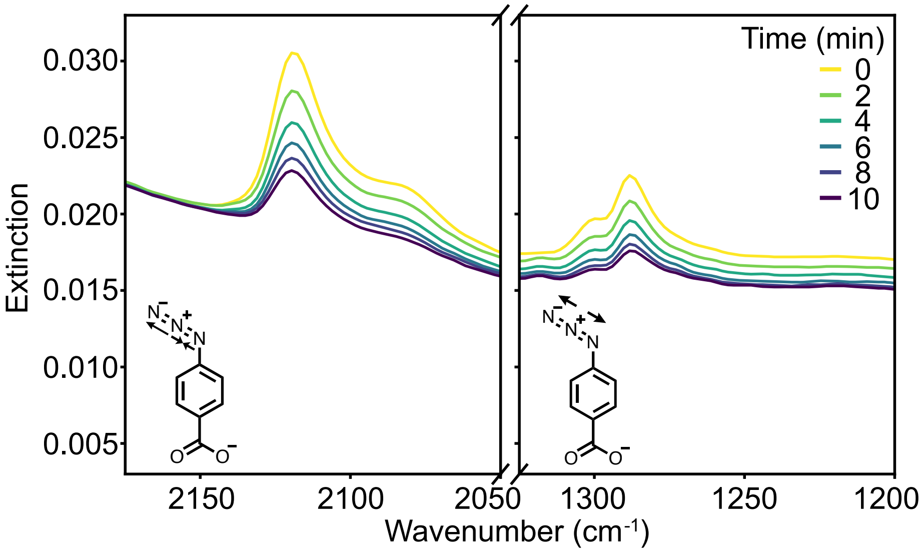

Figure S5: **The vibrational signal decays from the azide functional group.** We compared the signal decay of both the asymmetric stretching vibration (located around 2126 cm^{-1}) and the symmetric stretching mode (located around 1286 cm^{-1}), and found that the decay rate constants for both asymmetric (k_{asym}) and symmetric stretching (k_{sym}) modes are consistent.

Table S1: **The vibration signal decay kinetics of the asymmetric and symmetric stretching modes.** We investigated multiple Sn doping concentrations and found that the decay rate is the same within experimental error for both modes.

Sn %	k_{asym} (min^{-1})	k_{sym} (min^{-1})	k_{sym}/k_{asym}
0.5 %	0.097	0.080	0.82
0.9 %	0.23	0.22	0.96
2.0 %	0.16	0.18	1.13
3.3 %	0.11	0.10	0.91
7.0 %	0.12	0.12	1.00

5 Colloidal NC Properties

Table S2: **ITO NC doping and sizes measured from ICP-OES and SAXS, respectively from Figure 2a**

Sn (at%)	2.1	2.4	2.7
Diameter (nm), $2a$	11.2 ± 0.9	18.4 ± 1.4	25.6 ± 2.5

Table S3: **ITO NC doping and sizes measured from ICP-OES and SAXS, respectively from Figure 2c**

Sn (at%)	0.0	0.5	0.9	1.0
Diameter (nm), $2a$	26.2 ± 2.0	29.4 ± 4.0	28.2 ± 2.3	29.0 ± 2.3

Sn (at%)	2.0	2.3	3.3	7.0
Diameter (nm), $2a$	27.4 ± 3.2	27.0 ± 3.0	26.2 ± 2.2	27.8 ± 2.4

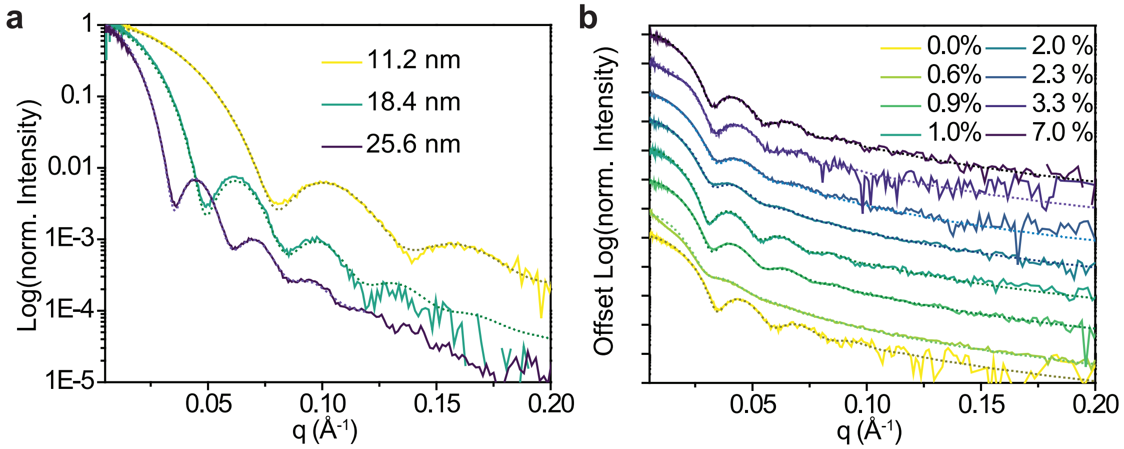

Figure S6: **Small-angle X-ray scattering patterns of ITO NCs dispersed in hexane for size characterization.** (a) ITO NCs with different sizes from Table S2. (b) ITO NCs with different Sn doping concentrations from Table S3. Note that in (b), after normalizing the logarithm of the scattered signal intensity, spectra were vertically offset to avoid overlap. The dotted lines indicate spherical form factor fits used to determine the NC core sizes.

6 Size Dependent Kinetics

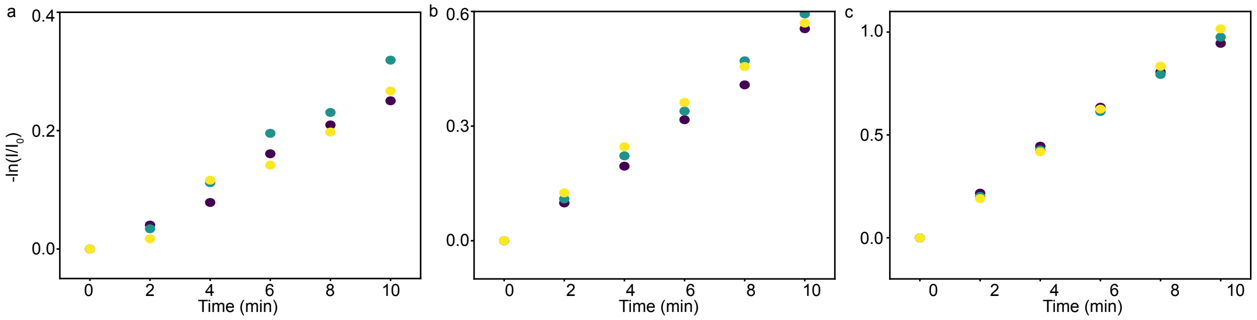

Figure S7: **First order kinetics of azide photolysis on different NC sizes** (a) 11.2 nm, (b) 18.4 nm, and (c) 25.6 nm sized ITO NC monolayers with their corresponding kinetics. Colors are arbitrary and indicate three replicate sets.

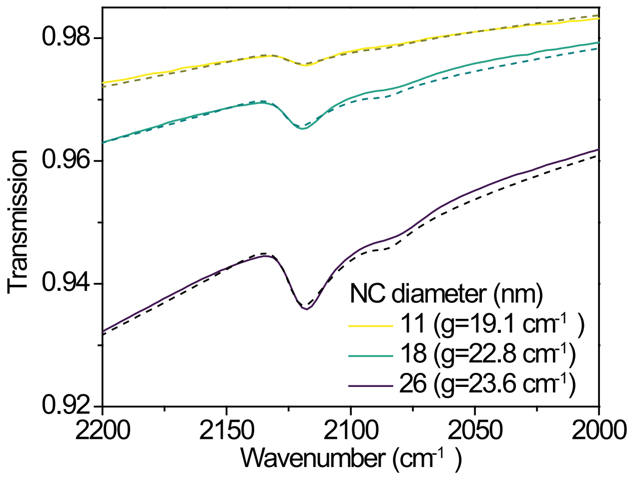

Figure S8: **Fitting of temporal coupled mode theory (TCMT) for ITO NCs of various sizes.** The legend indicates both the NC size and the coupling strength (g). We observe an increase in the g value with larger NCs. The TCMT model we used is the same as in our previous work.[1]

7 Detuning Dependent Kinetics

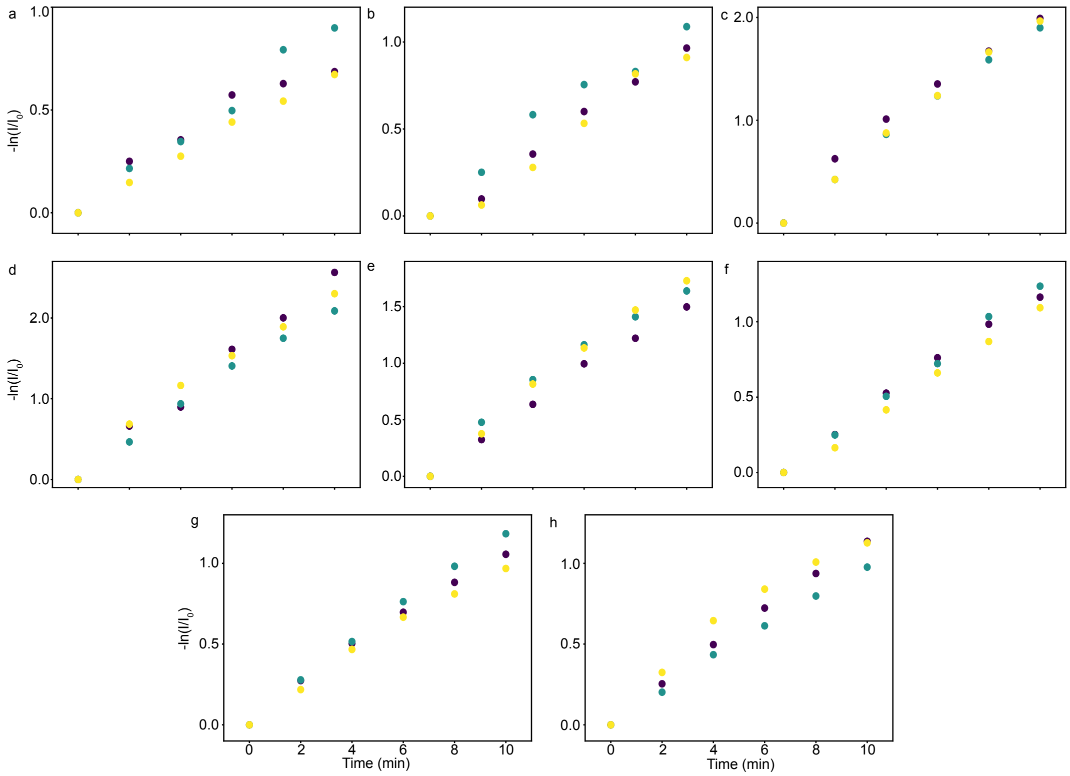

Figure S9: **First order kinetics of azide photolysis on differently doped NCs.** (a) 0% Sn, (b) 0.5% Sn, (c) 0.9% Sn, and (d) 1% Sn, (e) 2% Sn, (f) 2.3% Sn, (g) 3.3% Sn, (h) 7% Sn ITO NC monolayers with their corresponding kinetics. Colors are arbitrary and indicate three replicate sets.

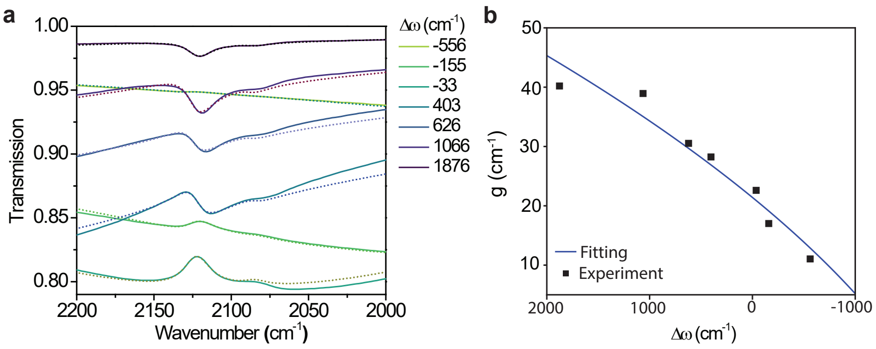

Figure S10: **TCMT fitting for ITO NCs with varying $\Delta\omega$.** (a) Spectra near the azide asymmetric stretching mode (solid lines) and corresponding TCMT fits (dotted lines). (b) Experimental (squares) and fitting (line) of the light-matter coupling strength (g) as a function of the detuning of cavity frequency with respect to the ground state vibrational frequency. We use a square-root fitting to describe the increase in the coupling strength as a function of the NC CPR frequency.[2]

8 UV-Vis Extinction Spectra

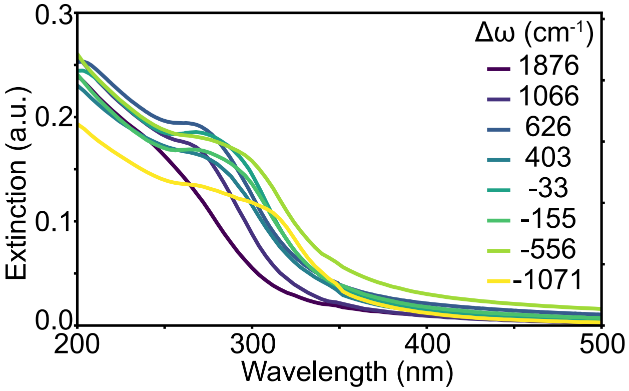

Figure S11: **UV-Vis extinction spectra of ITO NC monolayers after ligand exchange with ABzA on sapphire substrates with different Sn doping concentrations.** We used these spectra to generate Figure 3a.

Table S4: **ITO NC ML optical band gaps measured from UV-Vis data, respectively from Figure 3c**

Sn (at%)	0.0	0.5	0.9	1.0	2.0	2.3	3.3	7.0
BandGap (eV)	3.61	3.66	3.75	3.81	3.80	3.86	3.98	4.12

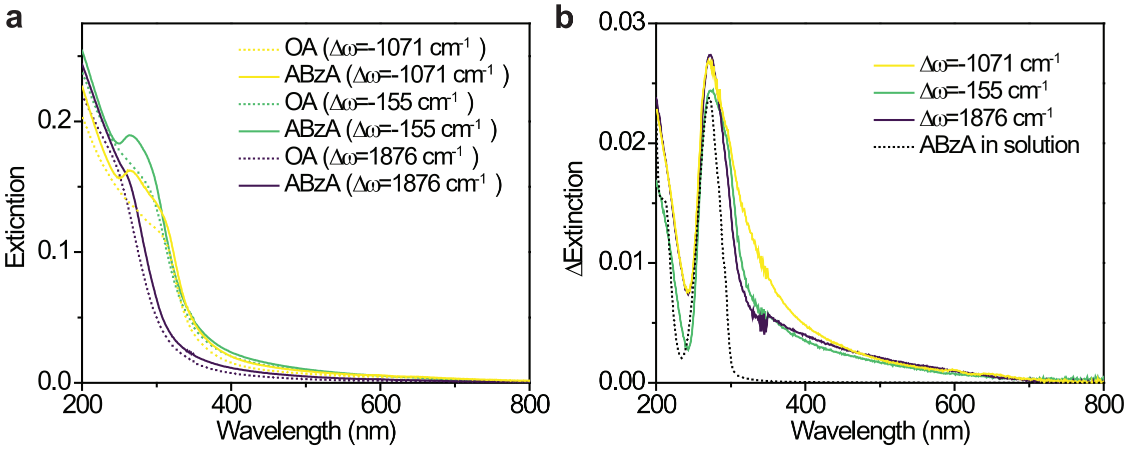

Figure S12: **UV-Vis extinction spectra of ITO NC monolayers before and after ligand exchange with ABzA molecules.** (a) UV-Vis spectra of ITO NC monolayers with different Sn doping concentrations (indicated by $\Delta\omega$) before (OA) and after (ABzA) ligand exchange. (b) Subtracted extinction spectra obtained by subtracting the pre-exchange signal from the post-exchange signal to isolate the ABzA absorption on ITO NCs. Note that the dotted black line represents the absorption spectrum of pure ABzA solution in acetonitrile.

9 Photolysis Rate and Extinction Spectra of ITO NCs on CaF_2 Substrates

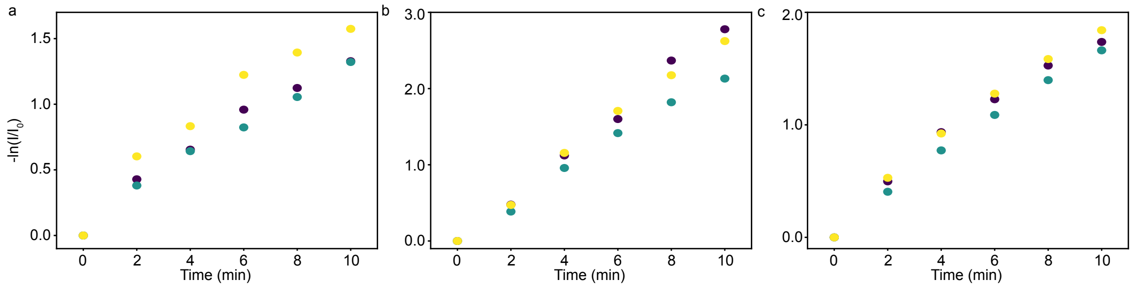

Figure S13: **First order kinetics of azide photolysis at different doping concentrations on CaF_2 substrates.** (a) 0% Sn, (b) 0.9% Sn, (c) 7.0% Sn doped ITO NC monolayers with their corresponding kinetics. Colors are arbitrary and indicate three replicate sets.

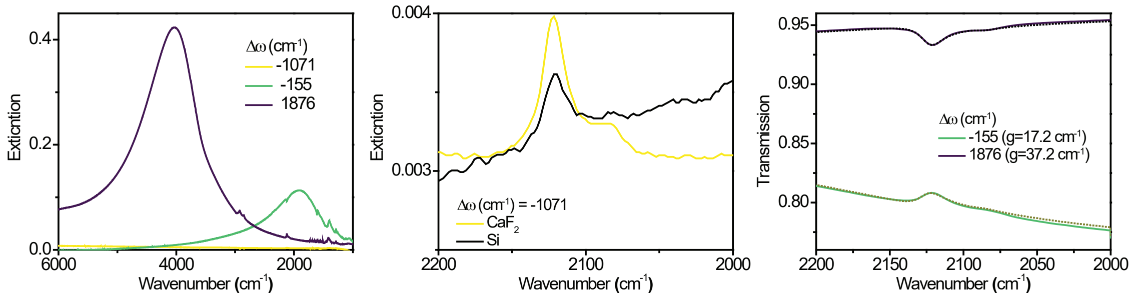

Figure S14: **Extinction spectra and vibrational intensity from ITO NC monolayers on CaF_2 substrates.** (a) Extinction spectra of ITO NCs with various $\Delta\omega$ on CaF_2 substrates. (b) Magnified extinction spectra of ITO NCs with $\Delta\omega = -1071$ cm^{-1} (0% Sn doped) on CaF_2 and Si substrates, highlighting the region near the azide asymmetric vibrational frequency. Each spectrum is normalized to that of the corresponding bare substrate. (c) TCMT fitting results for ITO NCs with $\Delta\omega = -155$ cm^{-1} and 1876 cm^{-1}.

10 Azide Molecules with Different Carbon Length

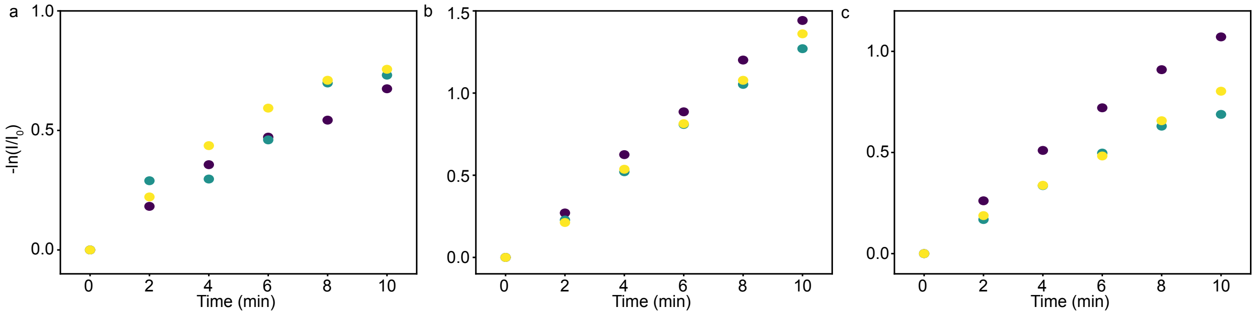

Figure S15: **First order kinetics from the azide functional group from (4-azidophenyl)acetic acid (C1 ABzA) molecules at different doping concentrations.** (a) 0% Sn, (b) 0.9% Sn, (c) 7.0% Sn doped ITO NC monolayers with their corresponding kinetics. Colors are arbitrary and indicate three replicate sets.

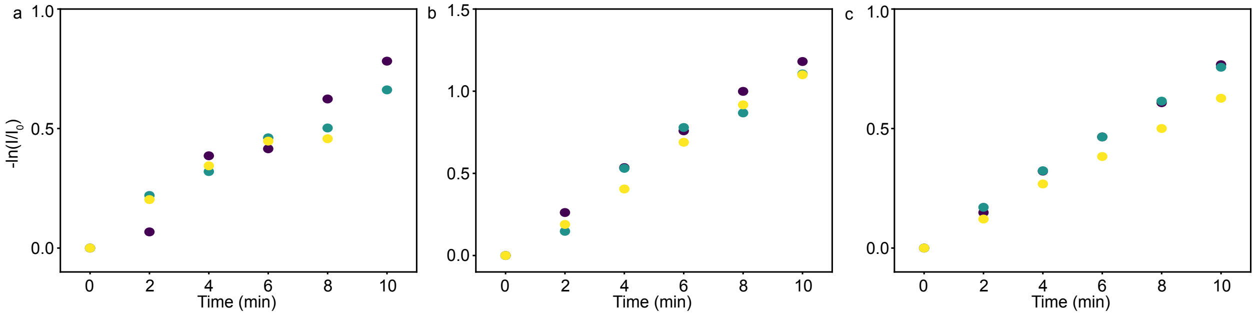

Figure S16: **First order kinetics from the azide functional group from 4-(4-azidophenyl)butyric acid (C3 ABzA) molecules at different doping concentrations.** (a) 0% Sn, (b) 0.9% Sn, (c) 7.0% Sn doped ITO NC monolayers with their corresponding kinetics. Colors are arbitrary and indicate three replicate sets.

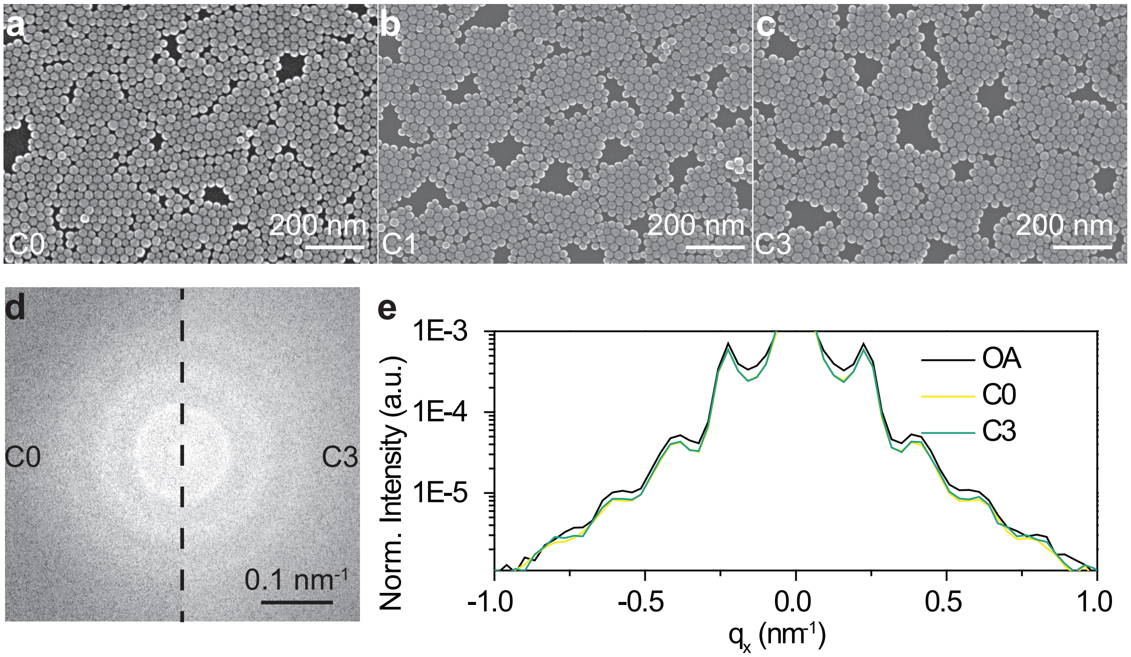

Figure S17: **SEM images and interparticle distances of ITO NCs.** SEM images of $\Delta\omega = -155$ cm^{-1} (0.9% Sn-doped) ITO NCs functionalized with (a) ABzA (C0), (b) C1 ABzA (C1), and (c) C3 ABzA (C3) molecules. (d) Fourier-transformed images of ITO NCs with C0 and C3 molecules. The estimated interparticle distance is approximately 1.8 nm. (e) Grazing incidence small-angle X-ray scattering (GISAXS) measurements of ITO NCs capped with oleate (OA), C0, and C3 ligands. A horizontal slice along the x-direction was taken to estimate interparticle distances, which are around 3.5 nm regardless of the ligand type.

11 Photolysis Rate for ITO NCs with Mixed Monolayers of ABzA and EBA

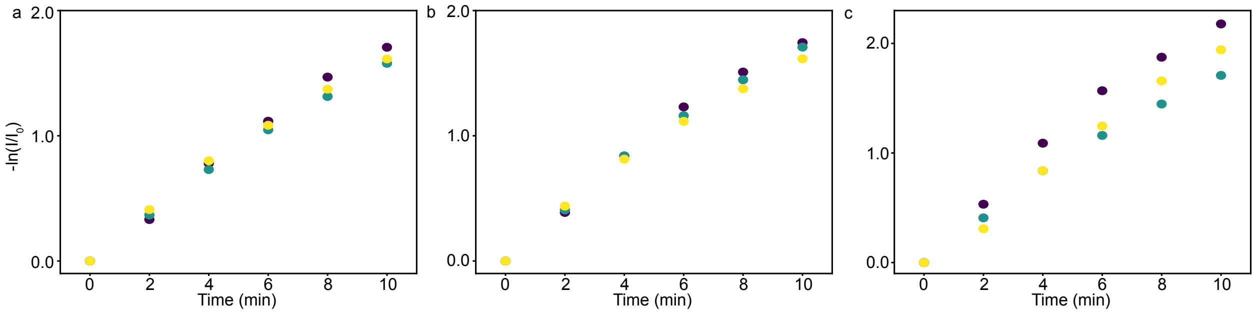

Figure S18: **First order kinetics from the azide functional group in mixed monolayers of ABzA and ethylbenzoic acid (EBA) at 0.9% Sn doped ITO NC monolayers.** (a) 30% ABzA, (b) 50% ABzA, and (c) 70% ABzA signals relative to NCs fully covered with ABzA on the NC surface. Colors are arbitrary and indicate three replicate sets.

12 Electronic structure calculations

All electronic structure calculations were performed in the gas phase using the Gaussian 16 software. Ground-state properties were computed using Density Functional Theory (DFT) with the ωB97X-D hybrid exchange–correlation functional and the def2-TZVP basis set. Excited-state properties were obtained at the same level of theory using Time-Dependent DFT (TD-DFT).

12.1 Features of UV-Vis absorption

To assess the photoactive excited states, the ABzA molecule was optimized in the ground state (S_0), and subsequent frequency calculations confirmed the structure as a minimum. Vertical excitation energies for the first six singlet states, along with their oscillator strengths, are summarized in Table S5. The results indicate that only S_2, S_3, S_5 and S_6 exhibit appreciable oscillator strengths. However, given the LED frequencies employed in the experiments, S_2 is identified as the relevant photoactive state following excitation.

Table S5: TD-DFT singlet excitation energies of the first 5 excited states at optimized ground state geometry (S_0). Results are shifted by 0.3 eV to match experimental data

State	Energy (nm)	Oscillator Strength
S_1	306.2	0.0003
S_2	272.0	0.4401
S_3	262.1	0.0599
S_4	250.5	0.0000
S_5	216.9	0.0828
S_6	206.6	0.1269

Figure S19 compares the experimental absorption spectrum of ABzA with the TD-DFT result. The TD-DFT spectrum is shown as vertical lines at the transition energies, with heights proportional to the corresponding oscillator strengths (Table S5). To facilitate comparison with the experimental linewidths, the spectrum was convolved with a Gaussian kernel (with standard deviation $\sigma = 0.2$ eV) to represent inhomogeneous/solvent broadening.

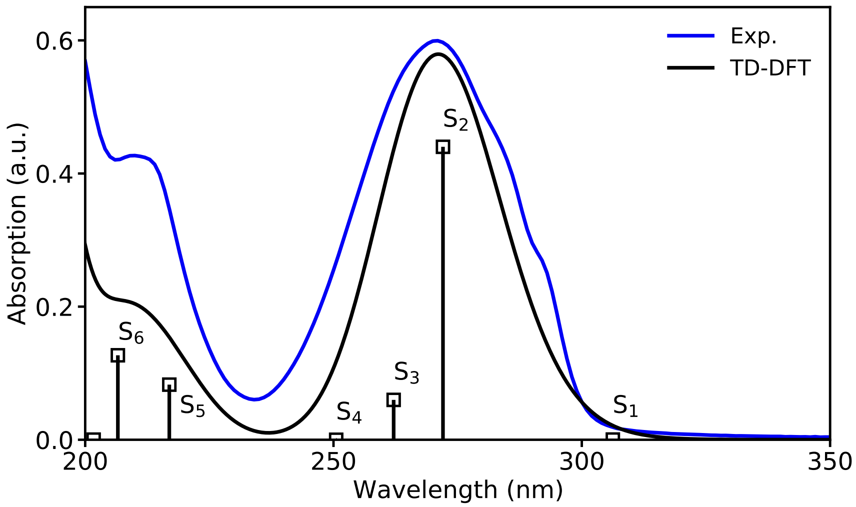

Figure S19: **Calculated UV-Vis absorption spectra of ABzA.** Experimental 0.33 mM ABzA in acetonitrile (blue) and TD-DFT (black) UV-Vis absorption spectra. Vertical lines represent the TD-DFT excitation energies, with the oscillator strengths. Each excitation energy was broadened with a Gaussian function (standard deviation $\sigma = 0.2$ eV), results are shifted by 0.3 eV to match experimental data.

12.2 IR frequencies

We focus on the vibrational frequencies associated with the $N-N$ dissociation in 4-azidobenzoic acid in the ground state (S_0) and their modification upon photoexcitation to the S_2 state. To obtain the IR frequencies, the molecule was optimized in both S_0 and S_2, followed by vibrational frequency analysis. The IR spectra for each state are presented in Figure S20. The calculated vibrational frequencies of the azide functional group for the S_0 and S_2 states are 2270.53 cm^{-1} and 2102.87 cm^{-1}, respectively. The optimized geometries, together with the atomic displacements corresponding to the fundamental modes, are shown in Figure 4 of the main text.

12.3 PES Scan

To investigate the dissociation dynamics of 4-azidobenzoic acid, we constructed the unrelaxed potential energy surface (PES) along the $N-N$ bond by incrementing the bond length by $\Delta x = 0.1$ Å from the ground-state optimized geometry. At each configuration, the energies

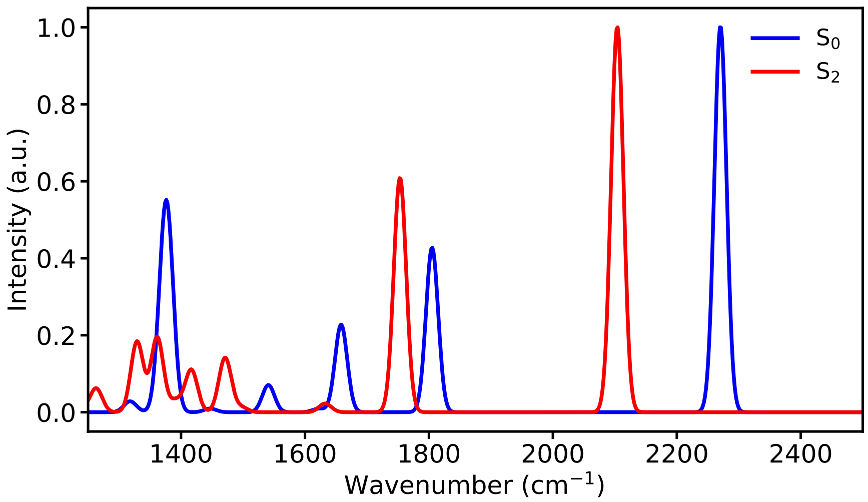

Figure S20: **Calculated IR absorption spectra of ABzA.** S_0 (blue) and S_2 (red) IR spectra. Each vibrational frequency was broadened with a Gaussian function (standard deviation $\sigma = 10$ cm^{-1}).

of the ground state and the first five singlet excited states were computed; the ground state and first three excited states are shown in Figure S21a. The PES indicates that the S_2 state is bound and does not lead to dissociation, whereas the S_1 state is dissociative. Thus, dissociation requires non-radiative relaxation from S_2 to S_1, consistent with previous studies on phenyl azides.[3,4] We used the Discrete Variable Representation (DVR) method to obtain the vibrational eigenstates of the S_2 surface. Figure S21b presents the close-up of the first three excited states, along with the first 10 vibrational states of the S_2 surface.

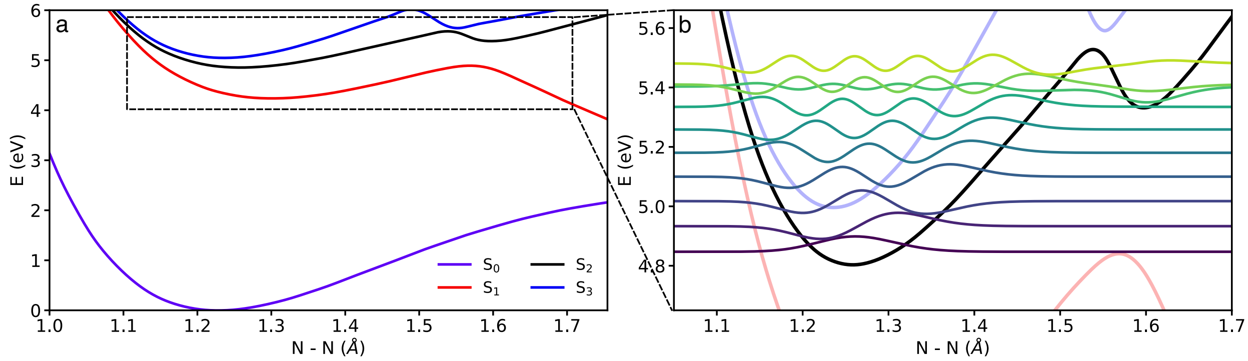

Figure S21: **Potential Energy Surface and Vibrational Structure of ABzA.** (a) Unrelaxed potential energy surfaces (PES) of the ground (S_0) and first three singlet excited states (S_1–S_3) along the N–N bond. (b) Close-up view of the S_2 surface showing the first ten vibrational eigenstates obtained using the Discrete Variable Representation (DVR) method.

13 Rate Theory

Here, we closely follow the derivation presented in Ref. 5. The molecule-cavity Hamiltonian is modeled as

$$\hat{H} = \hat{H}_{\mathrm{M}} + \hat{H}_c + \hat{H}_\nu + \hat{H}_{\mathrm{loss}} \tag{S1}$$

Where the molecular Hamiltonian $\hat{H}_{\mathrm{M}} = \frac{\hat{P}^2}{2M} + V(\hat{R})$ with M as the effective mass of the nuclear vibration and $V(\hat{R})$ as the electronic state potential energy surface. The light-matter interaction is expressed as:

$$\hat{H}_c = \frac{1}{2}\left[\hat{p}_c^2 + \omega_c^2\left(\hat{q}_c + \sqrt{\frac{2}{\omega_c}}\eta_c \cdot \mu(\hat{R})\right)^2\right] \tag{S2}$$

Here, $\hat{q}_c$ and $\hat{p}_c$ are the photon coordinate and momentum, respectively. ω_c is the cavity mode frequency, η_c is the light-matter coupling, and $\mu(\hat{R})$ is the dipole operator, which we assume to be linear and aligned with the cavity polarization such that $\mu(\hat{R}) \approx \hat{R}$.

$\hat{H}_\nu$ describes the phonon coupling to the molecular coordinate and $\hat{H}_{\mathrm{loss}}$ describes the cavity loss bath. Particularly, in the regime of low quality factors (Q), that is, low cavity lifetimes (τ_c), the cavity effects on the molecular coordinate are well described by performing a normal mode transformation where the cavity mode, and its associated loss bath can be included into an effective bath as:

$$\hat{H}_{\mathrm{eff}} = \hat{H}_c + \hat{H}_{\mathrm{loss}} = \frac{1}{2}\sum_\kappa\left[\hat{P}_\kappa^2 + \Omega_\kappa^2\left(X_\kappa - \frac{C_\kappa}{\Omega_\kappa^2}\hat{R}\right)^2\right] \tag{S3}$$

Here, the couplings (C_κ) and frequencies (Ω_κ) of these normal modes (X_κ) are sampled from the effective spectral density:

$$J_{\mathrm{eff}}(\omega) = \frac{2\tau_c^{-1}\eta_c^2\omega_c^3\omega}{(\omega_c^2 - \omega^2)^2 + (\tau_c^{-1}\omega)^2} \tag{S4}$$

And the Hamiltonian is rewritten as:

$$\hat{H} = \hat{H}_{\mathrm{M}} + \hat{h}_{B}^{\mathrm{eff}} + \hat{H}_{\mathrm{SB}} + \hat{H}_{\mathrm{ren}} \tag{S5}$$

Here

$$\hat{h}_{B}^{\mathrm{eff}} = \frac{1}{2}\sum_{i}(\hat{p}_i^2 + \omega_i^2 \hat{x}_i) + \frac{1}{2}\sum_{\kappa}(\hat{P}_\kappa^2 + \Omega_\kappa^2 \hat{X}_\kappa) \tag{S6a}$$

$$\hat{H}_{\mathrm{SB}} = -\hat{R} \otimes (\hat{F}_\nu + \hat{F}_{\mathrm{eff}}) \tag{S6b}$$

$$\hat{H}_{\mathrm{ren}} = (\lambda_\nu + \lambda_{\mathrm{eff}})\hat{R}^2 \tag{S6c}$$

Where $\hat{h}_{B}^{\mathrm{eff}}$ accounts for the effective and phonon bath energy, $\hat{H}_{\mathrm{ren}}$ is the bath reorganization energy and $\hat{H}_{\mathrm{SB}}$ is the system-bath interaction where we have defined $\hat{F}_\nu \equiv \sum_i c_i \hat{x}_i$ and $\hat{F}_{\mathrm{eff}} \equiv \sum_\kappa C_\kappa \hat{X}_\kappa$.

To provide a rate of vibrational excitation due to the cavity, we consider the Hilbert space spanned by the vibrational states $\{|\nu_n\rangle, |\nu_m\rangle\}$. In this subspace, the Hamiltonian of Eq. S5 can be expressed in the pseudo-spin representation as:

$$\hat{H}' = \hat{\mathcal{P}}\hat{H}\hat{\mathcal{P}} = \omega_0 \frac{\hat{\sigma}_z}{2} + \hat{\mathcal{P}} \otimes \hat{F}_{\mathrm{eff}} + \hat{h}_{B}^{\mathrm{eff}} + \Delta_x \hat{\sigma}_x \otimes \hat{F}_{\mathrm{eff}} + \epsilon_z \frac{\hat{\sigma}_z}{2} \otimes \hat{F}_\nu \tag{S7}$$

Here $\hat{\sigma}_z = |\nu_m\rangle\langle\nu_m| - |\nu_n\rangle\langle\nu_n|$, $\hat{\sigma}_x = |\nu_m\rangle\langle\nu_n| - |\nu_n\rangle\langle\nu_m|$, $\Delta_x = R_{nm} = \mu$ and $\epsilon_z = R_{mm} - R_{nn}$. Only the term proportional to Δ_x will cause vibrational transitions due to the cavity. The FGR rate for $|\nu_n\rangle \to |\nu_m\rangle$ transition inside the cavity can then be written as:

$$\begin{aligned} k_{\mathrm{VSC}} &= 2\int_0^\infty d\omega \; |\mu_{\mathrm{S}_2}|^2 J_{\mathrm{eff}}(\omega) \; \mathcal{A}_\nu(\omega - \omega_0) \cdot n(\omega) \\ &= 2|\mu_{\mathrm{S}_2}|^2 \langle\cos^2\theta\rangle \cdot \int_0^\infty d\omega \; \frac{2\tau_c^{-1}\eta_{\mathrm{c}}^2\omega_c^3\omega}{(\omega_{\mathrm{c}}^2 - \omega^2)^2 + (\tau_{\mathrm{c}}^{-1}\omega)^2} \; \mathcal{A}_\nu(\omega - \omega_0) \cdot n(\omega) \end{aligned} \tag{S8}$$

Here, $\langle\cos^2\theta\rangle \approx 1/3$ is the isotropic dipole angle orientation relative to the field polarization direction, τ_{c} is the plasmon-mode lifetime, and $n(\omega) = (e^{\beta\omega} - 1)^{-1}$ is the Bose-Einstein

distribution. The experimentally measured light-matter coupling, in the single molecule limit, can be expressed as $g_{\mathrm{c}}(\omega) = \eta_{\mathrm{c}}\omega_{\mathrm{c}} \cdot |\mu_{\mathrm{S}_0}|$, which can be experimentally extracted from Fig. S10. Eq S8 is then rewritten as

$$k_{\mathrm{VSC}} = 2\alpha\langle\cos^2\theta\rangle \cdot \int_0^{\infty} d\omega \; \frac{2g_{\mathrm{c}}^2(\omega)\tau_c^{-1}\omega_c\omega}{(\omega_{\mathrm{c}}^2 - \omega^2)^2 + (\tau_{\mathrm{c}}^{-1}\omega)^2} \; \mathcal{A}_\nu(\omega - \omega_0) \cdot n(\omega), \tag{S9}$$

where the parameter $\alpha = |\mu_{S_2}|^2/|\mu_{\mathrm{S}_0}|^2 = 0.3$ includes the ratio of the vibrational transition intensity, as well as the other approximation due to the use of FGR. It is expected that the light-matter coupling will have a square-root dependence on the cavity frequency,[2] therefore, we incorporate the observed cavity frequency-dependent behavior of $g(\omega)$ in our simulations by fitting the experimental data to a square root function $g(\omega) = a \cdot \sqrt{\omega} + b$, through the least-squares fitting procedure as implemented in the scipy package. The fitting and experimental data comparison is presented in Figure S10b with fitting parameters $a = 1.35$ cm$^{-1/2}$ and $b = -43.0$ cm^{-1}. Finally, $\mathcal{A}_\nu(\omega - \omega_0)$ is the broadening function of the excited state molecular vibrations, which we take as a Gaussian distribution centered around ω_0, where ω_0 is estimated from the vibrational normal mode frequency in the electronic excited state,

$$\mathcal{A}_\nu(\omega - \omega_0) = \frac{1}{\sqrt{2\pi\sigma^2}} \cdot \exp\left[-\frac{(\omega - \omega_0)^2}{2\sigma^2}\right] \tag{S10}$$

with $\sigma = 14.8$ cm^{-1}. In the main text, the rate enhancement is reported as:

$$k = k_0 + k_{\mathrm{VSC}}, \tag{S11}$$

where k_0 is the reaction rate constant outside the cavity.

13.1 Rates for different Carbon Chain Lengths

The light-matter coupling strength as a function of carbon chain length, evaluated at a detuning of $\Delta\omega \approx -133$ cm^{-1} (See Section S10) was fitted to an exponential decay model

$g = a \cdot e^{-b \cdot n_C} + c$, where n_C is the carbon chain length and fitting parameters $a = 1.11$ cm^{-1}, $b = 0.78$ and $c = 15.99$ cm^{-1}. The resulting coupling strengths were then used in Eq. S9 at the same detuning to compute the corresponding reaction rates. As shown in Figure S22b, the calculated rates exhibit a maximum variation of approximately 0.19 ($\sim 7\%$), indicating that the observed change in coupling strength has a moderate impact on the reaction rate under constant detuning conditions.

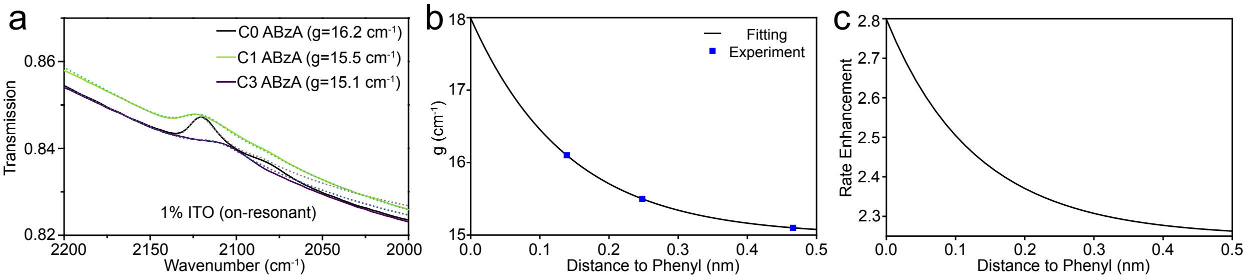

Figure S22: **Dependence of light-matter coupling and reaction rate on carbon chain length.** (a) Coupling strength (g) extracted from on-resonant ($\Delta\omega \approx -133$ cm^{-1}) ITO NCs functionalized with ligands having different carbon chain lengths between the carboxylate and phenyl groups, showing the rate enhancement trend in Figure 3c. (b) Experimental (squares) and fitted (line) light-matter coupling strength as a function of the carbon chain length. (c) Estimated FGR rate enhancement as a function of the carbon chain length. We saw that the rate enhancement is overestimated relative to the experiment shown in Figure 3c.

References

(1) Chang, W. J.; Roman, B. J.; Green, A. M.; Truskett, T. M.; Milliron, D. J. Surface-Enhanced Infrared Absorption Spectroscopy by Resonant Vibrational Coupling with Plasmonic Metal Oxide Nanocrystals. *ACS Nano* **2024**, *18*, 20636–20647.

(2) Mandal, A.; Taylor, M. A.; Weight, B. M.; Koessler, E. R.; Li, X.; Huo, P. Theoretical Advances in Polariton Chemistry and Molecular Cavity Quantum Electrodynamics. *Chem. Rev.* **2023**, *123*, 9786–9879.

(3) Das, S. K.; Odelius, M.; Banerjee, A. Simulating Non-Adiabatic Dynamics of Photoexcited Phenyl Azide: Investigating Electronic and Structural Relaxation en Route to the Formation of Phenyl Nitrene. *Chem. Eur. J.* **2024**, *30*, e202302178.

(4) Burdzinski, G.; Hackett, J. C.; Wang, J.; Gustafson, T. L.; Hadad, C. M.; Platz, M. S. Early Events in the Photochemistry of Aryl Azides from Femtosecond UV/Vis Spectroscopy and Quantum Chemical Calculations. *J. Am. Chem. Soc.* **2006**, *128*, 13402–13411.

(5) Ying, W.; Huo, P. Resonance theory and quantum dynamics simulations of vibrational polariton chemistry. *J. Chem. Phys.* **2023**, *159*.
